# The evolution of constitutive and induced defences to infectious disease

**DOI:** 10.1098/rspb.2018.0658

**Published:** 2018-07-25

**Authors:** Mike Boots, Alex Best

**Affiliations:** 1 Department of Integrative Biology, University of California, Berkeley, Berkeley CA, USA; 2 Department of Biosciences, University of Exeter, Penryn Campus, Penryn TR11 9FE, UK; 3 School of Mathematics and Statistics, University of Sheffield, Sheffield S3 7RH, UK

**Keywords:** immunity, defence, parasites, evolution, models, natural enemies

## Abstract

In response to infectious disease, hosts typically mount both constitutive and induced defences. Constitutive defence prevents infection in the first place, while induced defence typically shortens the infectious period. The two routes to defence, therefore, have very different implications not only to individuals but also to the epidemiology of the disease. Moreover, the costs of constitutive defences are likely to be paid even in the absence of disease, while induced defences are likely to incur the most substantial costs when they are used in response to infection. We examine theoretically the evolutionary implications of these fundamental differences. A key result is that high virulence in the parasite typically selects for higher induced defences even if they result in immunopathology leading to very high disease mortality. Disease impacts on fecundity are critical to the relative investment in constitutive and induced defence with important differences found when parasites castrate their hosts. The trade-off between constitutive and induced defence has been cited as a cause of the diversity in defence, but we show that the trade-off alone is unlikely to lead to diversity. Our models provide a framework to examine relative investment in different defence components both experimentally and in the field.

## 1. Introduction

Defence against infectious disease results from a complex set of interdependent mechanisms ranging from mechanical barriers through to the complex array of effectors in the immune system [1,2]. This complexity has ultimately been shaped by epidemiology and evolution and we, therefore, need to understand how the costs and benefits of the production, maintenance, and use of different immune components determine the optimal immune response in hosts with a range of life histories challenged with a diverse range of infectious diseases [2,3]. An important distinction is that between defence mechanisms that prevent full-blown infection in the first place (constitutive) and those that act after infection (induced). Constitutive defences include mechanical barriers such as in animals, the skin and gut walls in addition to preformed antimicrobials in vertebrates [2], the phenyloxydase cascade in invertebrates [4], alongside numerous plant toxins that generally act constitutively [5]. Induced defences are activated once infection has occurred, being typified by mechanisms such as reactive oxygen species, cytokines, and antibodies, which are elicited upon infection in vertebrates [2], antimicrobial peptides in invertebrates [6], and the hypersensitive response in plants [7]. These two forms of defence have often been discussed in response to herbivory [8–10] and in the context of extreme induced anti-predation modifications in invertebrates such as the spines of Daphnia [11], but they are found across vertebrates, invertebrates, plants, and microbial systems in response to infectious disease [2,12]. These two arms of the immune system have obvious differences in terms of individual health because, in the case of induced defences, individuals are infected for a time and may suffer disease symptoms in addition to immunopathology. Less intuitively, there are also important implications to population-level impacts of these two classes of defence.

The distinction between constitutive and induced defence is important to the individual because if infection is prevented through constitutive defence, there is no damage caused by the parasite directly and little risk of damage due to the use of the immune system (immunopathology). However, it is important to understand that the distinction also has fundamentally different population-level epidemiological impacts: constitutive defence prevents onward infection, while induced defence will only shorten the infectious period. As a consequence, in order to understand the optimal use of constitutive against induced defence, we need to take into account epidemiological impacts and population dynamics as well as individual impacts of the relative investment in the two forms of defence. We know that organisms have both constitutive (always present) and induced (activated at infection) defence mechanisms to infectious disease, but we lack a clear understanding of what determines the relative investment and diversity of these two arms of the immune system.

The key to this understanding is not only the effectiveness and costs of the immune component for the individual but also the population-level impact on the epidemiology of the disease such as the prevalence or the force of infection [13]. Clearly, the defence mechanism has an impact on the spread and ultimately the prevalence of the infectious disease, and this feedbacks onto the selection for defence in the first place with important implications to optimal defence and diversification [13–16]. Eco-evolutionary theory [17] allows us to understand how the interplay of individual costs and benefits of different forms of defence and the particular ecological feedbacks that they cause determine the optimal level and diversity of defence that we see in nature.

Another important distinction between constitutive and induced defence is the way in which costs to defence are likely to be manifested. Without costs whether they are associated with either the development and maintenance or the use of the defence mechanism, we would clearly expect maximal defence in all individuals. There is considerable empirical evidence that higher resistance to disease is associated with fitness costs in terms of life-history characters such as development time or competitive ability [18–20]. These costs make intuitive sense given the costs of production and maintenance of even basic defences such as thicker mechanical barriers [21] and the fact that we see considerable variation in defence both within and between species in nature [22–24]. In addition, it is well established experimentally that there are costs of using induced defence mechanisms [25]. Although induced defences may come with some production and maintenance costs in the absence of disease, it is a useful generalization that the key costs for induced defences are manifested only upon their use, but that there are costs in the absence of disease to constitutive defences [26].

Here, we develop theory that makes general predictions on how both host and parasite characteristics have an impact on the relative investment in constitutive against induced defence in the context of both the impact on the individual and the population-level feedback on the epidemiology of the disease. There has been theory on the evolution of inducible defences to predators and herbivores [27–32] and now a number of models in the context of infectious disease [26,31,32]. However, these models do not take into account population-level effects of constitutive and induced defence and the consequential ecological feedback on selection. Rather they focus only on the contrasting implications of the two forms of defence on individuals. We, therefore, lack a general theory that examines optimal investment in constitutive and induced defence that takes into account both their individual and population-level impacts. Here, our main aim is to examine how relative investment in constitutive and induced defence varies with the epidemiological characteristics of the infectious disease involved and the life-history characteristics of the host. There has also been interest, particularly in plant systems, in the idea that a trade-off between constitutive and induced defence may explain the diversity in defence strategies within and between species [9]. There is evidence for this trade-off in nature [9], but here we examine whether such a trade-off can lead to diversity in defence through evolutionary branching.

## 2. Modelling

We make the simplifying assumption that constitutive defence mechanisms are costly in terms of trade-offs with other life-history parameters—evolutionary costs—while costs of defence post infection are manifested in the costs due to the use of the immune system. In our models, we define *constitutive* defence as reduced susceptibility to infection (avoidance). Both susceptible and infected hosts are assumed to pay the cost of the constitutive defence, which occurs through a reduced reproductive rate. We define *induced* defence as an increased ability to clear disease (an increased recovery rate). Only infected hosts pay the cost of an induced defence, which is an increase in mortality while infected (immunopathology). These assumptions are deliberately simplistic, because there may be costs in the absence of disease to induce defences, and the costs of use of the immune system may be manifested in reduced fitness once recovered [33]. However, this simplicity allows us to develop a baseline model from which future work that includes more complex assumptions about costs can be developed. Our simplifying assumptions do, however, mean that constitutive costs are expressed on fecundity, while induced costs act on mortality. We could assume that constitutive costs act again only in the absence of disease but on natural mortality such that *β* = *f*(*b*) and that induced costs only occur on action but on fecundity *γ* = *f*(*f*), and thereby allow both costs to act either on mortality or fecundity while maintaining the distinction between when costs act. This would allow us to theoretically examine the impact of this additional difference between the way we have modelled constitutive and induced defence, that the costs in one acts on fecundity, while the other acts on mortality. However, there is no evidence of trade-offs with constitutive resistance and natural mortality, and action costs of immunity on fecundity while infected have not been measured. Sublethal costs on fecundity for recovered individuals may be immunopathological and further theoretical work will examine the impact of these different costs, in the context of coevolutionary models.

To allow us to explore the epidemiological feedbacks to the evolution of the different defence mechanisms, we base our analysis on a classic compartmental epidemiological model:
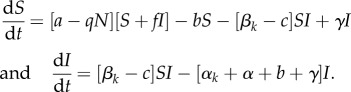
All individuals are born susceptible at rate *a*, which is reduced due to crowding by a factor *qN* (where *N* = *S* + *I*). Infected hosts potentially suffer a reduced birth rate by sterilizing factor *f* (when *f* = 0, the parasite is a castrator) and all hosts die at rate *b*. Transmission is a density-dependent mass-action process, with a constant part (*β_k_*) and a part limited due to constitutive defence, *c*. Infected hosts suffer increased mortality, also made up of a constant part (*α_k_*) and an immunopathological part, *α*. Finally, infected hosts can recover at rate *γ*.

We conduct our analysis within the evolutionary invasion framework of adaptive dynamics [34,35]. As such, we assume that a rare ‘mutant’ host strain, with a small phenotypic difference in a trait, attempts to invade a ‘resident’ host strain that has reached a population dynamic attractor of the epidemiological model. The success of the mutant depends upon its invasion fitness, defined as its growth rate when rare. For the most part we assume that the constitutive and induced defences evolve independently of each other, meaning that we can treat our model as coevolutionary. We will assume that constitutive defence incurs costs to the birth rate (such that *a* is a function of *c*) and that induced defence incurs immunopathological costs (such that *α* is a function of *γ*). By considering the stability of the resident–mutant equilibrium, the fitnesses of each mutant type (either with a mutation in the level of constitutive or induced defence) can then be given by
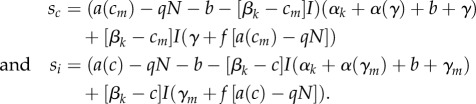
The two host strains will evolve in the direction of their local selection gradients until it reaches an ‘evolutionarily singular point’ where there is no longer directional selection on either trait. We shall also look at cases where the two types of defence are directly traded off against one another (i.e. *γ* is a function of *c*), both with and without further life-history costs (see electronic supplementary material).

Our focus is on how investment in constitutive and induced defence varies across ecological and epidemiological gradients. From an adaptive dynamics perspective we are, therefore, interested in *Continuously Stable Strategies* (CSS), an attracting and uninvadable endpoint of evolution. Theory has shown that, generally, such points exist, provided that evolving increased defence incurs accelerating costs [36,37]. We, therefore, choose our two trade-offs to take the following forms in order that there is an optimal defence that we can examine and the singular points are scaled between 0 and 1:
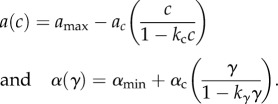
In both cases, the costs of defence are accelerating. Assuming these trade-offs, the co-CSS of investment in constitutive and induced defence for a particular parameter set can be found by plotting the two CSSs against one another. Where these two lines cross will be a coevolutionary attracting point (we note that this condition is neither necessary nor sufficient, and in particular it is possible for both strategies to be at an individual CSS that is a coevolutionary repeller [38]. For all of the examples here we checked that our results gave true co-CSS points). We also examined the outcome under individual evolution of the two traits, but there were no qualitative, only quantitative, differences in the outcomes.

## 3. Results

### (a) Determinants of relative investment in constitutive and induced defence against parasites

#### (i) Disease characteristics

**Figure 1.**
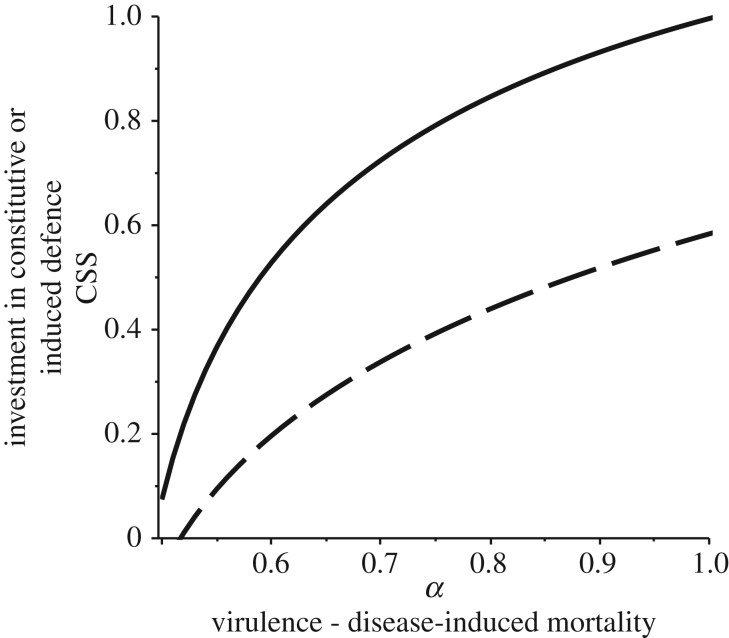
Plots of the optimal (continuously stable) strategy in investment in constitutive (solid line) and induced (dashed line) defences against parasite virulence where the main effect of the parasite is on death rather than fecundity. We see an increase in both arms of the immune system as the virulence (*α*) of the disease increases. Default parameter values are *β_k_* = 2, *q* = 0.2, *b* = 1, *α_k_* = 0.6, *f* = 1, *a*_max_ = 11, *a_c_* = 0.02, *k_c_* = 0.65, *α*_max_ = 0.99, *α_c_* = 0.02, *k_γ_* = 0.65.

In response to parasites that cause higher virulence (disease-induced mortality) there is selection for investment in both higher constitutive and induced defences (figure 1*a*). A key implication of this is, given the immunopathological costs of induced defence, the result of higher investment in induced defence is that individuals have a very high chance of death due to the combined effects of high virulence from the pathogen and strong immunopathological costs. Naturally virulent diseases are, therefore, doubly problematic from an individual host's point of view because they not only kill you faster but may select for stronger self-harm. Overall, the ratio of investment in constitutive and induced defences remains fairly constant when virulence increases (see electronic supplementary material). There are relatively minor impacts of the parasite transmission rate and minor impacts on fecundity investment in constitutive and induced defence (results not shown).

#### (ii) Host characteristics

**Figure 2.**
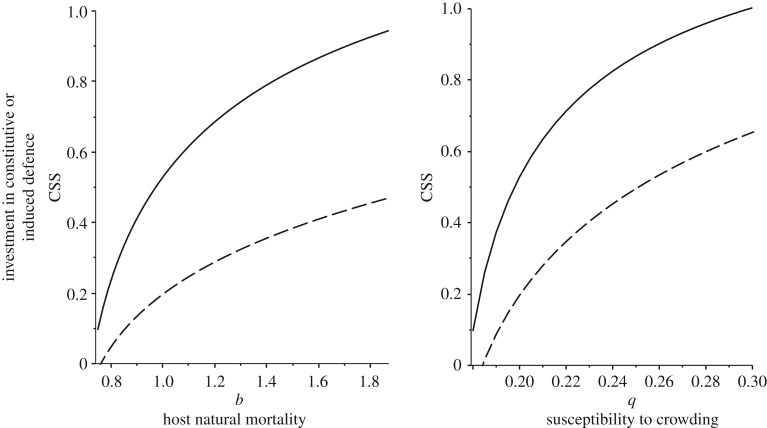
Plots of the optimal (continuously stable) strategy in investment in constitutive (solid line) and induced (dashed line) defences against different host characteristics. In (*a*) investment increases particularly in constitutive defences as hosts are shorter lived (higher background mortality (*b*)), while in (*b*) investment increases and levels off as susceptibility to crowding increases (*q*). Default parameter values are the same as figure 1.

When we look at characteristics of the host (figure 2), we see that there is a pattern of decreasing investment in both arms of the immune system with lifespan (i.e. 1/mortality; figure 2*a*) and a lower susceptibility to crowding (figure 2*b*). Lower susceptibility to crowding means denser populations characteristic of pest species and our models show that the ratio of investment in the two arms remains fairly constant as overall investment falls (figure 2*b*). With lifespan, although investment in both arms falls in longer-lived organisms (lower death rate *b*, figure 2*a*), there is a faster fall in constitutive defence such that short-lived organisms invest relatively more in constitutive rather than induced defences (figure 2*a*). For these parameters the overall investment in immunity falls as lifespan increases. This somewhat counter-intuitive result is parameter-dependent, but has been found in similar models that assume only evolutionary costs to constitutive defence [39]. Longer lifespan can lead to lower investment in models with epidemiological feedbacks due to the balance of paying the costs for a long time, high chances of infection, and in particular the ability to still reproduce when infected [39]. With our model the range of parameters where overall immunity decreases with lifespan is wider than in baseline models because the costs of induced defence are paid for by increased virulence and, therefore, costs to resistance become relatively stronger in longer-lived organisms.

### (b) Determinants of relative investment in constitutive and induced defence against castrators

There can be a fundamental distinction between parasites that castrate the host and those that allow reproduction in terms of the ecological feedbacks that they create and, therefore, how they impact disease resistance [40,41]. As a consequence, although we have examined the impact of the reduction in fecundity as an individual trait, it is important to examine the impact of host and disease traits on the two arms of the defence system when the parasite castrates the host.

#### (i) Disease characteristics

**Figure 3.**
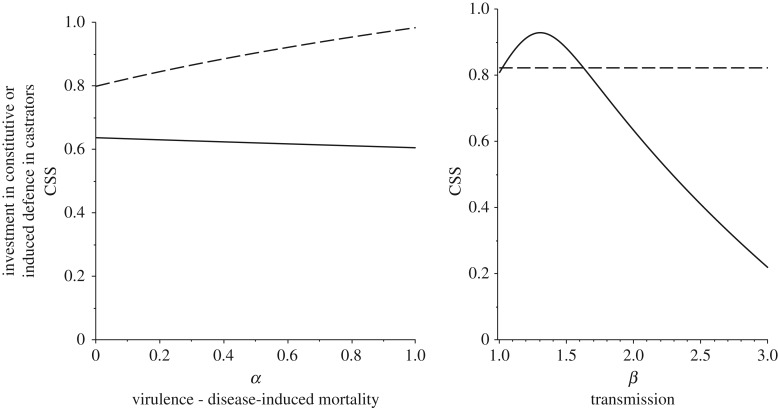
Plots of the optimal (continuously stable) strategy in investment in constitutive (solid line) and induced (dashed line) defences against different parasite characteristics for castrators (*f* = 0). In (*a*), we see relatively little effect of parasite virulence on constitutive defences, but again increased induced defences as alpha increases, in (*b*), constitutive defences peak and then fall as the transmission rate increases with no effect on induced defence. Default parameter values are as in figure 1 except: *α_k_* = 0.1, *a_c_* = 2.5, *k_c_* = 0.4, *α*_max_ = 0.1, *α_c_* = 2, *k_γ_* = 0.4.

In castrators, virulence has little impact on investment in constitutive defence, but higher virulence selects for higher induced defences (figure 3*a*). As such, high virulence again leads to relatively higher investment in induced defence despite its immunopathological costs, leading to more lethal disease interactions due to parasites with high virulence selecting for defence responses that lead to higher mortality. With transmission rate, generally induced defences are rather insensitive to transmission rates. By contrast, constitutive defences peak in investment at relatively low-to-intermediate transmission rates (figure 3*b*) due to low initial risk of infection when transmission is very low and the high costs of defence increasingly not been worth paying when transmission becomes higher. These effects are generally rather minor.

#### (ii) Host characteristics

**Figure 4.**
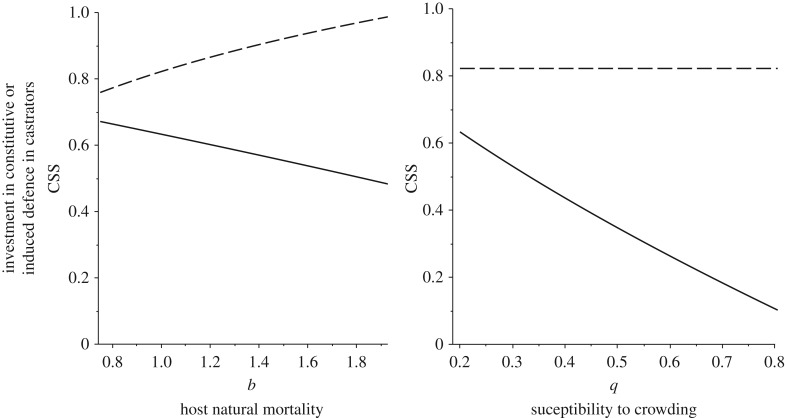
Plots of the optimal (continuously stable) strategy in investment in constitutive (solid line) and induced (dashed line) defences against different host characteristics when the parasite is a castrator (*f* = 0). In (*a*), shorter-lived organisms invest less in induced and more in constitutive defence, while in (*b*), as susceptibility to crowding (*q*) increases, there is selection for reduced constitutive defence but induced defences are unaffected. Default parameter values are as in figure 3.

In response to castrators, investment in induced defence decreases in longer-lived hosts (i.e. low death rate), while constitutive defence investment increases (figure 4*a*). We would, therefore, predict that longer-lived organisms invest relatively more in constitutive rather than induced defences that are costly to use when faced with castrators. Hosts with relatively low self-limitation, characteristic of pest species, will invest relatively more in constitutive and less in induced defences when faced with castrators, while more self-limited species are predicted to invest in induced rather than constitutive defences (figure 4*b*). More generally, the prediction is that hosts with high population densities will invest in constitutive defences, while hosts at low densities will rely more on induced defences. Higher densities selecting for constitutive defence (see electronic supplementary material, [42]) is consistent with classic optimal defence theory based on studies of defence against herbivory [43], but only when the infectious disease are castrators. Herbivores and predators act ecologically in a similar way to castrators when there is no recovery which explains the similarity of the results.

### (c) The generation of diversity: trade-off between constitutive and induced defences

So far, we have assumed that constitutive and induced defences are evolutionarily independent. However, there is increasing evidence that across a range of systems there is a trade-off between constitutive and induced defences. This trade-off has been suggested to be an explanation for the diversity in host defence both within and between species. Trade-offs are a compelling process by which coexistence can occur because different genotypes or species can use different combinations of constitutive and induced defence in this case to achieve the same outcome. However, this argument often relies on an assumption of equalizing trade-offs such that all the different genotypes have exactly the same fitness. Robust coexistence, however, requires the ability to invade each other from rare and can be examined by the potential for evolutionary branching in evolutionary ecological models [35,44]. The potential for evolutionary branching in the model depends on there being ‘mutual invadability’ at a singular point 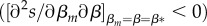 such that there can be a protected polymorphism. However, if we assume a direct trade-off between induced (*c*) and constitutive (*γ*) defence, with no further life-history costs to either defence mechanism, we find that 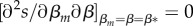, meaning that evolutionary branching and coexistence is not generated by a trade-off between induced (*c*) and constitutive (*γ*) defence in our model (see electronic supplementary material). As such, this trade-off alone is unlikely to generate and maintain diversity in defence traits apart from in the case of coexistence of genotypes with equal fitness.

If in addition to a trade-off between induced and constitutive defence there is a cost to higher defence *per se* (either through higher constitutive defence leading to lower birth rate or through higher induced defence having higher immunopathological costs as we have assumed earlier), there is the potential for evolutionary branching leading to protected polymorphisms (we find that necessarily 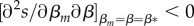). We would then see diversity with genotypes coexisting that are trading off constitutive and induced defence such that there are individuals with high constitutive and low induced defence or *vice versa*. However, an important insight is that this is only likely when there are in addition costs to higher defence: the simple trade-off between the two arms of the defence system is not sufficient.

## 4. Discussion

We have examined how optimal investment in constitutive (always present and costly) as opposed to induced (activated and costly only on infection) immunity is determined by host and disease characteristics. Fundamental to our approach is that we consider the selection that is due to not only the direct effects on the individual but also the impact of the different defences on the epidemiology of the disease. Moreover, we assume that the two defence mechanisms have different types of costs: constitutive defences are always present and, therefore, costly in the absence of disease while, in contrast, costs occur only when induced defences are used in response to infection. It is important to note that there is a fundamental emergent second cost to induced defence that is the damage to infection before the immune response occurs. Both the nature of the disease and the characteristics of the host determine the optimal investment. A key result is that in response to a highly virulent parasite, induced defences are favoured even though that results in immunopathology leading to extremely high infected mortality. There is a clear distinction between parasites that castrate their hosts and those that don't, and we show that the trade-off between constitutive and induced defence is unlikely on its own to generate and maintain diversity.

We show an increased investment in induced defences in response to more virulent (lethal) disease. In the case of castrators, the relative investment in induced rather than constitutive defence also increases in response to more virulent parasites. This is an important result because it follows from this that because induced defences are assumed to be costly through immunopathology, virulent diseases become doubly harmful, both through the damage they cause directly and the immunopathology of the immune system that they select for. Critically, this suggests that the observed pathology of highly virulent disease may be a consequence, in part, of the way in which they favour investment in induced defence. Intuitively, we may think that in response to a highly lethal disease there would be selection against the use of an immune defence that causes immunopathology, but the opposite is true due to epidemiological feedbacks that alter prevalence and, therefore, the risk of infection.

The characteristics of the host also determine the investment in constitutive and induced defences. In castrators, longer-lived hosts are selected to invest relatively more into constitutive rather than induced defences than short-lived hosts. For longer-lived organisms, there is more chance of being challenged during a longer life and, therefore, more reason to invest in a constitutive defence. However, against non-castrators, the pattern reverses, with constitutive defences relatively higher in shorter-lived hosts. In general, there is a clear distinction in the qualitative outcomes between castrators and more classical diseases that only increase the death rate of their hosts that results from our explicit consideration of the ecological feedbacks to selection on defence. In particular, the relationship between virulence and prevalence of the disease in the population is different in castrators [40,44,45]. For both castrators and non-castrators longer-lived hosts are less likely to invest in a strong induced defence. A longer-lived host would on average pay the costs of a strong immune defence on more occasions due to the risk of multiple challenges and as such induced defence may be less favourable to a long-lived host. However, to balance this, there is also more chance of mortality or lost fecundity due to infection before the induced defence activates. Our models show that generally the balance between these processes leads to a relatively small effect.

We show that there is overall less investment in defence in hosts with high carrying capacities. Moreover, particularly in response to castrators, we show that relatively fast, high carrying capacity pest species will invest more in constitutive than induced defences. This relationship has been demonstrated experimentally in a bacterial phage laboratory system that possesses an induced defence based on CRISPR [42]. At higher densities in better medium, constitutive defence was selected for, while at lower-density CRISPR, the induced defence was much more common [42]. We, therefore, have some experimental data that are consistent with this prediction in castrators, but there is the need for more comparative and experimental data in a range of systems.

A key hypothesis to explain the immense variability both within and among species in defence strategies is that there is a trade-off between constitutive and induced defence [9]. Although the data have been mixed as a whole, a large-scale recent study has provided strong evidence for this trade-off across a range of plant species [9]. We have not assumed this trade-off in our general models, but with this assumption (either with or without further life-history costs) the results are qualitatively the same. We have shown that this trade-off alone is not sufficient for the generation and maintenance of diversity through evolutionary branching and is, therefore, not likely to lead to stable polymorphism. Such diversity can only arise if there are further costs of defence to life history. The existence of a trade-off *per se* may allow a form of neutral diversity with exactly equal fitness for a range of species or genotypes along the trade-off, but this form of equalizing trade-off is less likely to lead to persistence of diversity in the face of stochasticity and genetic drift [46]. Another important message of our work is that we have not assumed a trade-off between constitutive and induced defence, but given that often the two defence mechanisms can be selected in opposite directions, it may appear that there is a trade-off. As such, the relationship seen in nature may actually result from these selective pressures and the correlated costs of the two arms of the immune system rather than a direct trade-off between them.

Throughout, we have made the assumption that induced defences leading to recovery are only costly while infected. Such costs have been shown directly particularly in insect experiments [25] and are well studied in terms of the immunopathology of many important human diseases [47]. Our assumption that constitutive defences are costly in terms of other life-history traits in the absence of disease is also well supported [2,20,21,48]. Clearly, it is possible that both defences may have elements of both types of costs and that the costs of use of the immune system may be manifested in reduced fitness through fecundity or mortality effects for recovered individuals, but the distinction in the nature of the costs for different defence mechanisms is a useful simplification. The fact that we assume different costs for the two defences is a key driver of our results and gives our model its novelty compared to previous papers on the evolution of different forms of defence (i.e. [39,49,50]). There may be components of the induced system that have less significant or even insignificant costs to use; however, many of the insights from our models still hold if only constitutive defence is costly in terms of host life history. The reason for this is that the key cost of induced defence is the emergent cost of being infected before the defence mechanism kicks in. This is the fundamental cost of induced defence and the key driver of selection on this arm of the defence system. As discussed in the model set-up, we have also assumed that costs to constitutive defence act on fecundity, while those on induced defence impact mortality, and in particular have not examined the impact of immunopathology on the reproductive output of recovered individuals. Further work examining the interplay of the defence arms and different cost structures would be very useful particularly in the context of specific infectious disease systems.

Existing theoretical work on inducible defences in nature has tended to focus on predator–prey [30,51] or plant–herbivory systems [27], concentrating on the importance of variability in the environment in selecting for induced defences. We have assumed constant environments with differences in equilibrium population size and prevalence emerging from the epidemiological feedbacks, but variable environments may well have important implications for selection on the two arms of defence. In some systems castration may not occur instantly and this delay may reduce the differences that we show here between castrators and classic parasites, and clearly recovery may not lead to complete restoration of reproduction. Another important simplification that, because we assume challenge by a single strain of one parasite, we have implicitly assumed that there is no difference in the specificity of induced and the constitutive defence and no variability in the parasite. Constitutive and induced defences are likely to vary significantly in their specificity [2] and this is likely to have a significant impact on their evolution. Previous theory on the induced and constitutive defence against infectious disease has emphasized the importance of variability in the parasite and uncertainty over the dose and growth rate of the infectious agent [31,52]. These previous models on infectious disease suggest that a higher probability of infection selects for greater relative investment in constitutive responses, but their focus is on the optimal combination of induced and constitutive defence in the context of uncertain infection risk and virulence [31,52]. It is, therefore, important to extend our current theory to examine the impact of variability in the parasite and also to include parasite coevolution. Moreover, different forms of defence are known to create different selective pressures on parasite virulence [53] and, therefore, it would be an interesting next step to look at coevolution in response to induced and constitutive defence. We also make the assumption that there is no specificity in defence such that all parasite genotypes are equally defended. Such specificity has been shown to be important in host defence in general [16] and in a recent model that examines the optimal allocation of induced and constitutive defence but without epidemiological feedbacks [26]. Therefore, a general model with both specificity and epidemiological feedbacks is an important next step towards a full understanding of induced and constitutive defence.

We have made a series of predictions of the impact of host and disease characteristics on relative investment in constitutive and induced defence. Epidemiological feedbacks on the evolutionary outcome are critical and this work emphasizes that a full understanding cannot be gained without considering the population as well as the individual impacts of different disease threats and host responses. The fundamental difference in the nature of the costs is also critical to the outcome and it is important to emphasize that the emergent costs of induced defence is critical to both the individual and these population-level impacts. Our predictions need experimental testing and as more comparative data become available, we will better understand their relevance in nature. We have not included uncertainty and variability in the environment or the infectious disease threat, but still show that there are key differences in the selection pressures for constitutive and induced defences in nature.

## Supplementary Material

Supplementary materials

## Data accessibility

There are no data associated with the study.
