# Supplementary material for: The evolution of constitutive and induced defences to infectious disease
Source: Proc Biol Sci. 2018 Jul 25;285(1883):20180658. doi: 10.1098/rspb.2018.0658 (PMC6083258; doi:10.1098/rspb.2018.0658)
Supplement: Supplementary materials [file rspb20180658supp1.pdf]

<u>Online Supplementary Information for "The evolution of constitutive vs induced defense to infectious disease" by Boots & Best</u>

Derivation of fitness

Given recovery and infected reproduction, direct calculation of the mutant invasion fitness is difficult. Here we derive fitness proxies by determining when a resident equilibrium loses stability to a mutant invader. In particular, we consider a four-dimensional system of ordinary differential equations consisting of resident susceptible and infected types and rare mutant susceptible and infected types. We then consider the equilibrium at which the resident is at its standard equilibrium S^*, I^* and the mutant is absent $S_m \approx 0, I_m \approx 0$. The stability of this equilibrium, (S^*, I^*, S_m, I_m), depends on a 4x4 Jacobian matrix of partial derivatives.

Since we know the resident equilibrium is stable in the absence of the mutant, and the resident equations are independent of mutant densities, the stability depends entirely on the 2x2 sub-matrix of the mutant's equations, for example for the constitutive defence case,

$$J = \begin{pmatrix} a(c_m) - qN^* - b - [\beta_k - c_m]I^* & \gamma + f[a(c_m) - qN^*] \\ [\beta_k - c_m]I^* & -(\alpha + b + \gamma) \end{pmatrix}$$

For the resident-mutant equilibrium to be unstable, this Jacobian must yield at least one positive eigenvalue. It can be found that this is necessarily the case whenever the determinant is negative, yielding the condition,

$$s = \left(a(c_m) - qN - b - [\beta_k - c_m]I\right)\left(\alpha + b + \gamma\right) + [\beta_k - c_m]I\left(\gamma + f[a(c_m) - qN]\right) > 0.$$

This is necessarily sign equivalent to the true fitness, and we therefore take this to be the fitness proxy.

Constitutive – Induced trade-off with no further life-history costs

We assume a trade-off between *c* and γ with no further relationships with other traits in the host's life-history. The population dynamics are as in equation (1)-(2) in the main text, and the fitness is now given by,

$$s = \left(a - qN - b - [\beta_k - c(\gamma_m)]I\right)\left(\alpha + b + \gamma_m\right) + [\beta_k - c(\gamma_m)]I\left(\gamma_m + f[a - qN]\right)$$

Evolutionary branching

Provided a singular point exists that is mutually invadible, with mixed second derivative, $\left[\partial^2 s / \partial\gamma_m \partial\gamma\right]_{\gamma_m=\gamma} < 0$ then a trade-off can always be chosen which allows for evolutionary branching. In this model we find that,

$$\left.\frac{\partial s}{\partial \gamma_m}\right|_{\gamma_m=\gamma} = 0 \Rightarrow c'(\gamma) = -\frac{a - qN - b}{I\left[\alpha + b - f\left(a - qN\right)\right]}$$

and,

$$\left.\frac{\partial^2 s}{\partial \gamma_m \partial \gamma}\right|_{\gamma_m=\gamma} = -qN'(\gamma) + c'(\gamma)I'(\gamma)\left[\alpha + b - f\left(a - qS - 2qI\right)\right] + c'(\gamma)S'(\gamma)fqI$$

When the necessary derivatives are substituted in to this expression, it in fact reduces to $\left[\partial^2 s / \partial\gamma_m \partial\gamma\right]_{\gamma_m=\gamma} = 0$. As such, no trade-offs exist that allow for evolutionary branching assuming this trade-off.

Investment patterns

We also present here some corresponding plots to those in the main text showing how investment varies with the model parameters. We still plot results for both constitutive and induced defence here (figure S1), but reiterate that the

two are directly linked by the trade-off. For these results we assumed a trade-off of,

$$c(\gamma) = 0.4 + 0.2\left(\frac{1-\gamma}{1-0.5\gamma}\right)$$

which guarantees a continuously stable strategy at all points tested. We note that now $c \in [0.4, 0.6]$. Here we see that investment in constitutive (solid line) increases with both virulence and mortality. We also find that investment is constant as both sterility and competitive ability are varied.

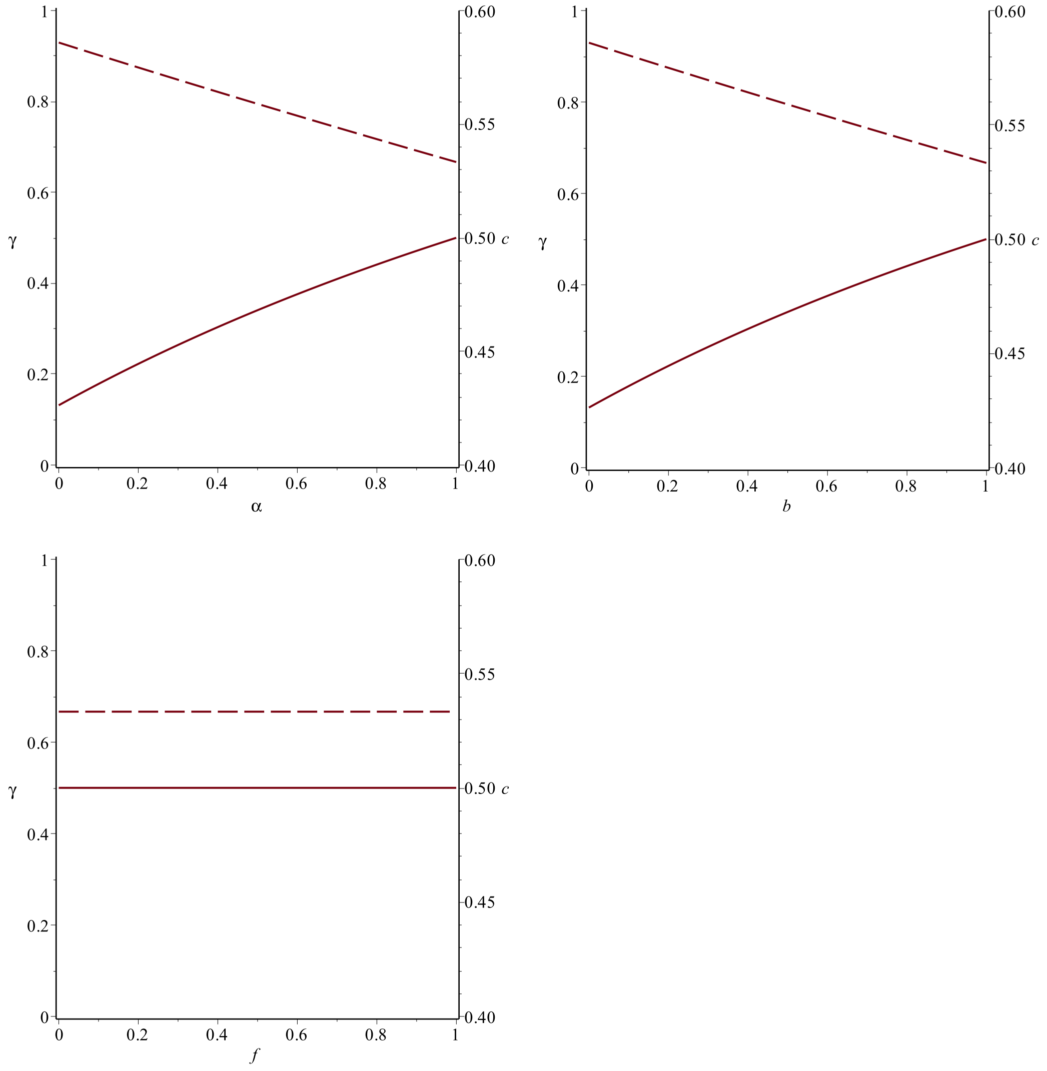

Figure S1 – Patterns of investment in constitutive (solid, right y-axis) and induced (dashed, left y-axis) line when there is a direct trade-off between the two traits as virulence, death rate and fecundity are varied. Parameter values are as of figures in main text.

Constitituve – Induced trade-off with further life-history costs

Now we assume a trade-off between c and γ but with further costs to the host's life-history. Here we will assume that the birth rate, a, is also linked to the defence traits with $c(\gamma)$ and $a(\gamma)$. The population dynamics are as in equation (1)-(2) in the main text, and the fitness is now given by,

$$s = \left(a(\gamma_m) - qN - b - [\beta_k - c(\gamma_m)]I\right)\left(\alpha + b + \gamma_m\right) + [\beta_k - c(\gamma_m)]I\left(\gamma_m + f[a(\gamma_m) - qN]\right)$$

Evolutionary branching

Provided a singular point exists that is mutually invadible, with mixed second derivative, $\left[\partial^2 s / \partial\gamma_m \partial\gamma\right]_{\gamma_m=\gamma} < 0$ then a trade-off can always be chosen which allows for evolutionary branching. For analytical ease let us assume that *f=0*. In this case we find that,

$$\left.\frac{\partial s}{\partial \gamma_m}\right|_{\gamma_m=\gamma} = 0 \Rightarrow c'(\gamma) = -\frac{a - qN - b}{I\left[\alpha + b\right]} - a'(\gamma)$$

and,

$$\left.\frac{\partial^2 s}{\partial \gamma_m \partial \gamma}\right|_{\gamma_m=\gamma} = -\frac{q(\alpha+b+\gamma)^2\left(q[\alpha+b+\gamma]+[\alpha+b]\left[\beta_k - c(\gamma)\right]\right)\left[a'(\gamma)\right]^2}{(\alpha+b)\left(q[\alpha+b+\gamma]-[a(\gamma)-b]\left[\beta_k - c(\gamma)\right]\right)^2}$$

after the necessary derivatives are substituted in. This term is necessarily negative for any trade-off $a(\gamma)$, meaning that now evolutionary branching can occur. Numerical exploration suggests that this result also holds for $f>0$.

Investment patterns

We also present here some corresponding plots to those in the main text showing how investment varies with the model parameters. We still plot results for both constitutive and induced defence here, but reiterate that the two are directly linked by the trade-off. For these results we assumed trade-offs of,

$$c(\gamma) = 0.4 + 0.2\left(\frac{1-\gamma}{1-0.5\gamma}\right)$$
$$a(\gamma) = 4.01 - 0.02[\gamma + c(\gamma)]$$

which guarantees a continuously stable strategy at all points tested. The latter trade-off can be understood as causing a greater cost to the birth rate when both forms of defence are high, although the change to the birth rate is only very slight in this example. Again we note that now $c \in [0.4, 0.6]$. The trends are shown in figure S2. Here increasing virulence causes a non-linear response, initially reducing investment in constitutive defence (as in the main model) before increasing investment at high levels, while there is increased constitutive defence at high mortality rates. We see low investment in constitutive defence against sterilizing parasites (as in the main model). We also have a non-linear response with competitive ability, with initially decreasing investment in constitutive (as in the main model) but increasing investment at high levels of competition.

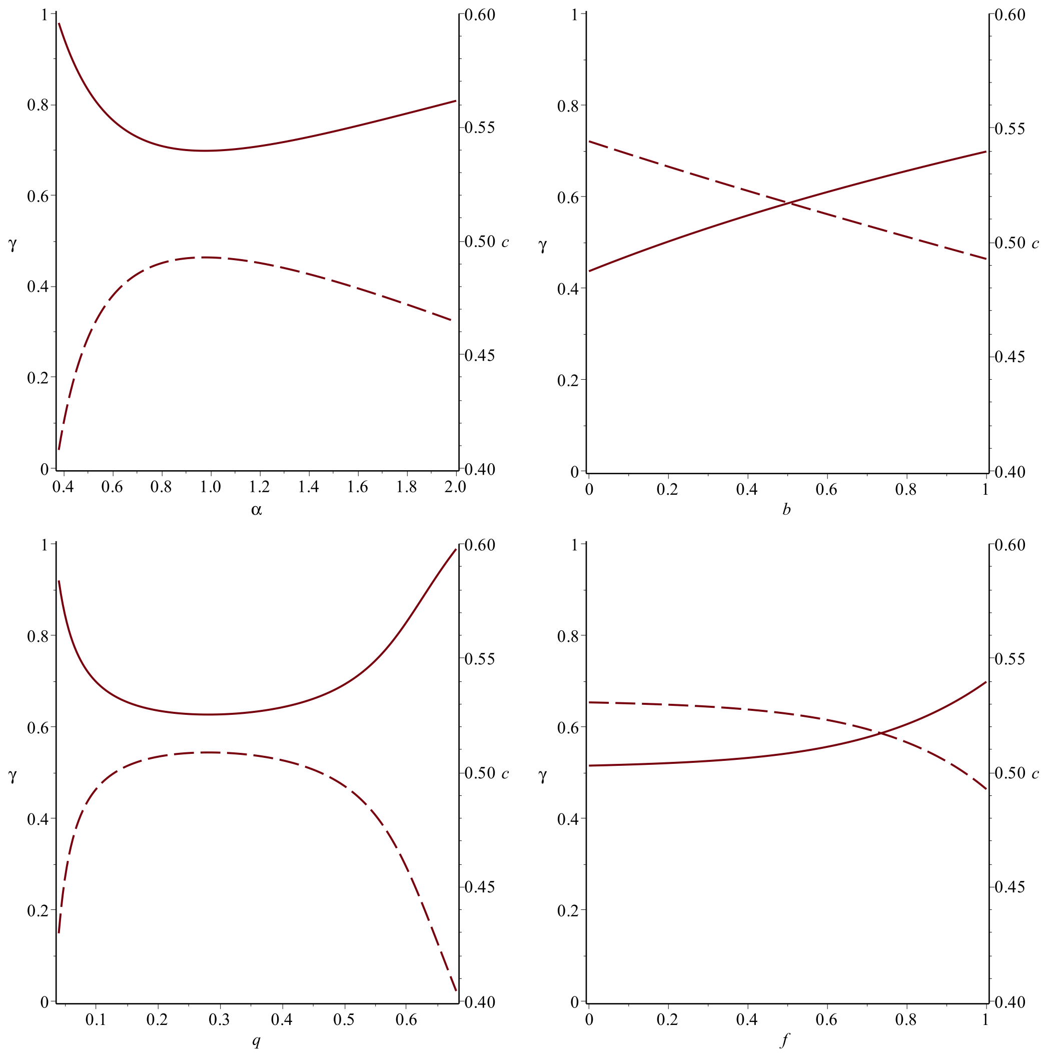

Figure S2 – Patterns of investment in constitutive (solid, right y-axis) and induced (dashed, left y-axis) line when there is a direct trade-off between the two traits and an additional cost to the birth rate, as virulence, death rate, competition and fecundity are varied. Parameter values are as of figures in main text.

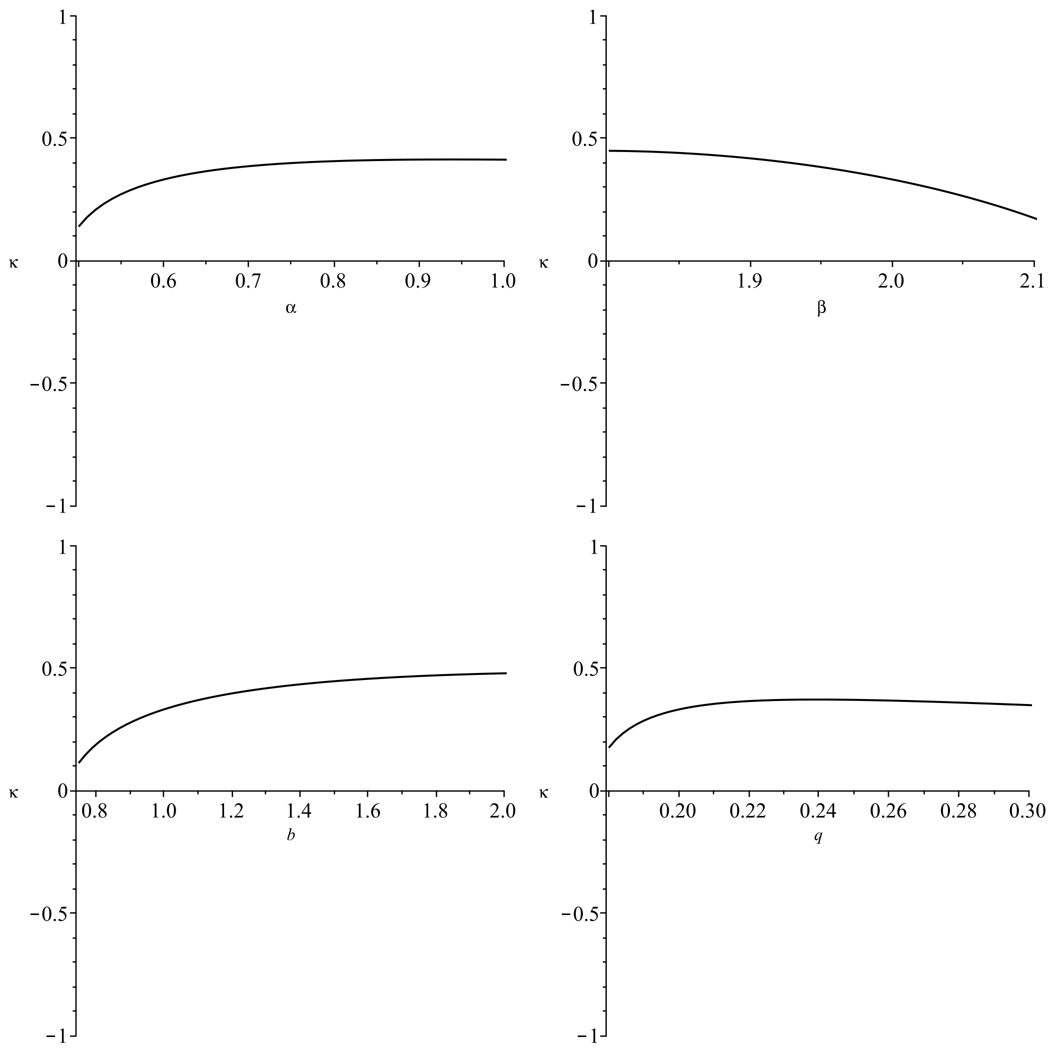

Figure S3

Plots showing the differential in investment $\kappa = c - \gamma$ as model parameters are varied, without castration (c.f. figures 1 and 2). These show that there is increased relative investment in constitutive defence at high death rates, low transmission rates and intermediate competition levels.

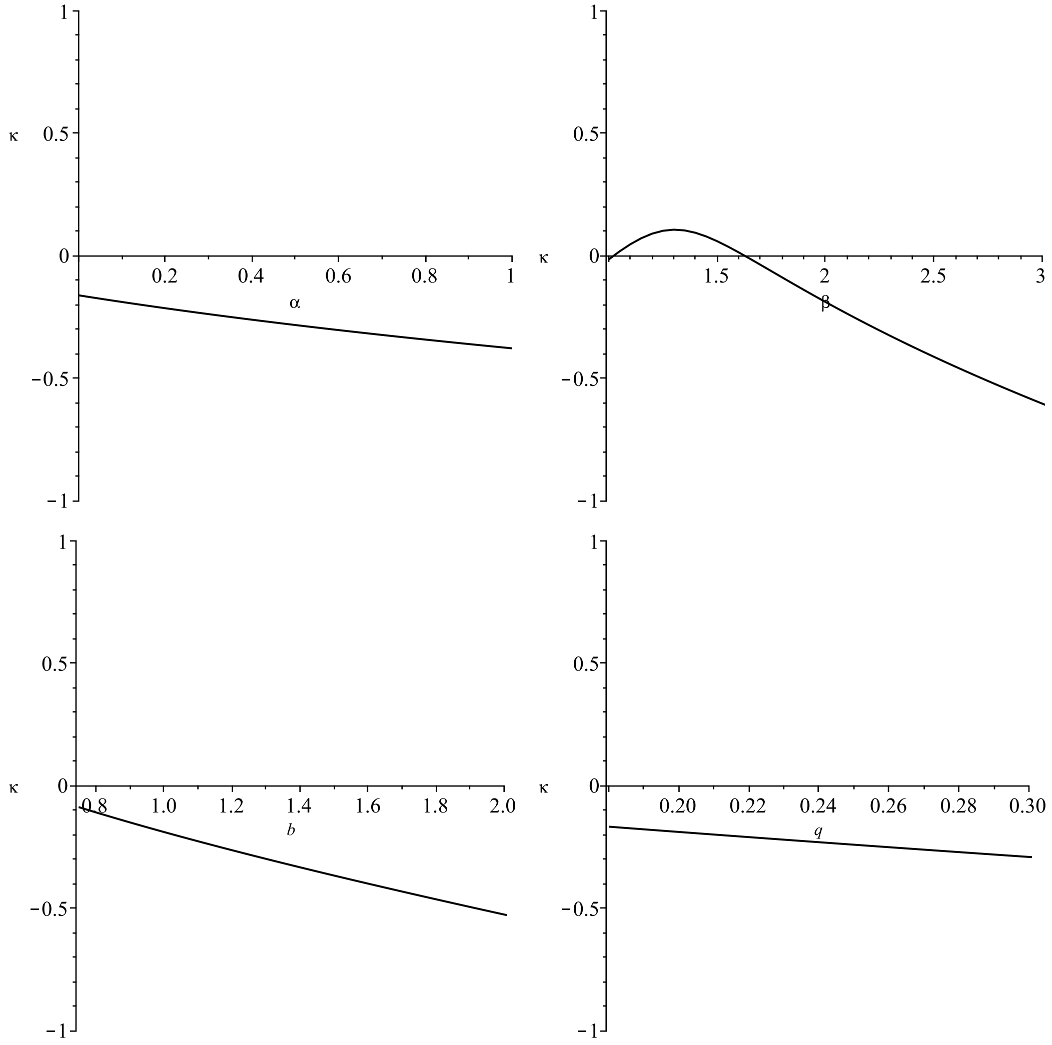

Figure S4

Plots showing the differential in investment $\kappa = c - \gamma$ as model parameters are varied, with castration (c.f. figures 3 and 4). Contrary to the previous figure, these show increased relative investment in induced defence at high death rates. The pattern with transmission is now non-monotonic.

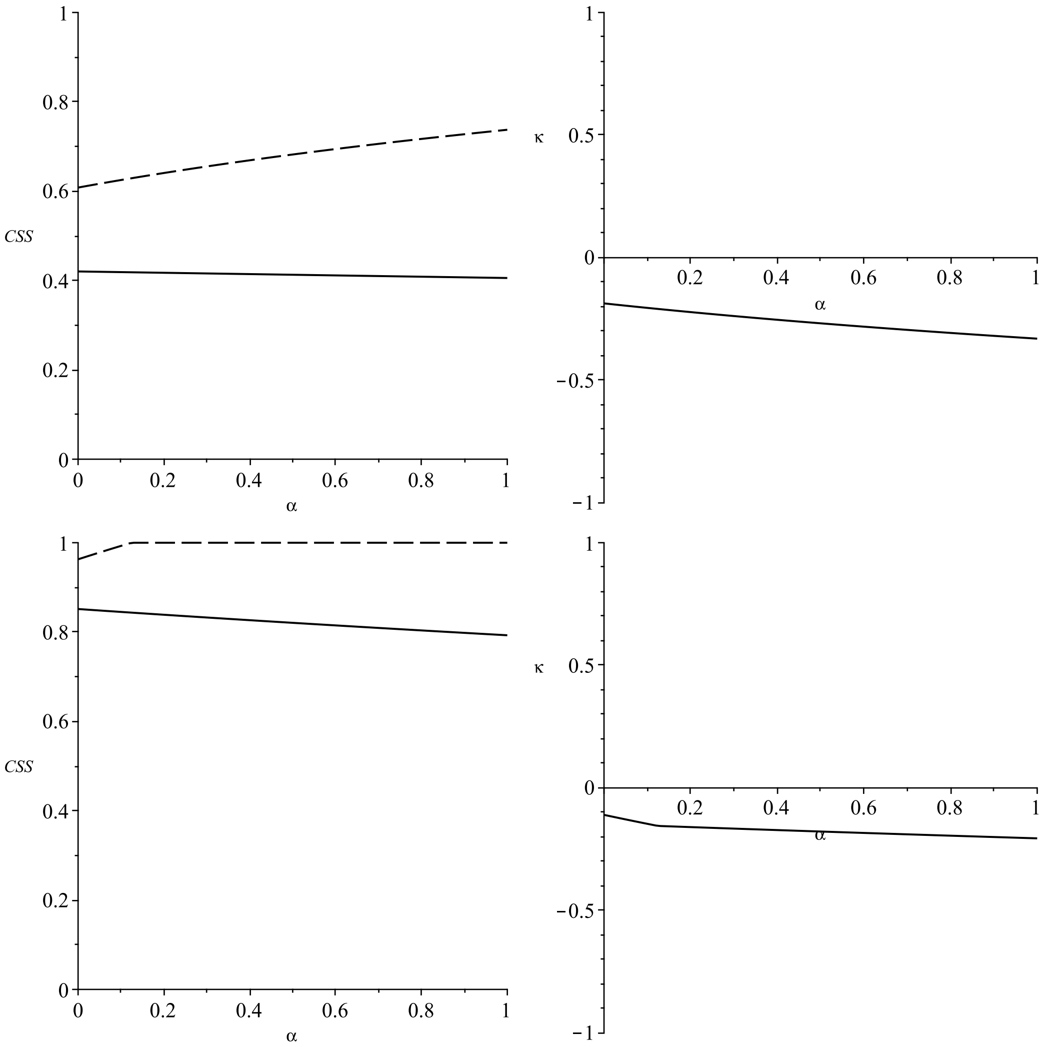

Figure S5

Plots showing investment as virulence, α, is varied for different trade-off functions to the main text, with castration (c.f. figure 3a). Top-row $k_c = k_\gamma = 0.6$ (higher curvature), bottom-row $k_c = k_\gamma = 0.3$ (lower curvature), with parameters otherwise the same as figure 3. These plots demonstrate that a higher (lower) trade-off curvature shifts the investment down (up) but the broad pattern remains the same. In the bottom-left plot, once investment reaches $\gamma = 1$ this is the maximum bound of the trade-off, and investment is then maximized.

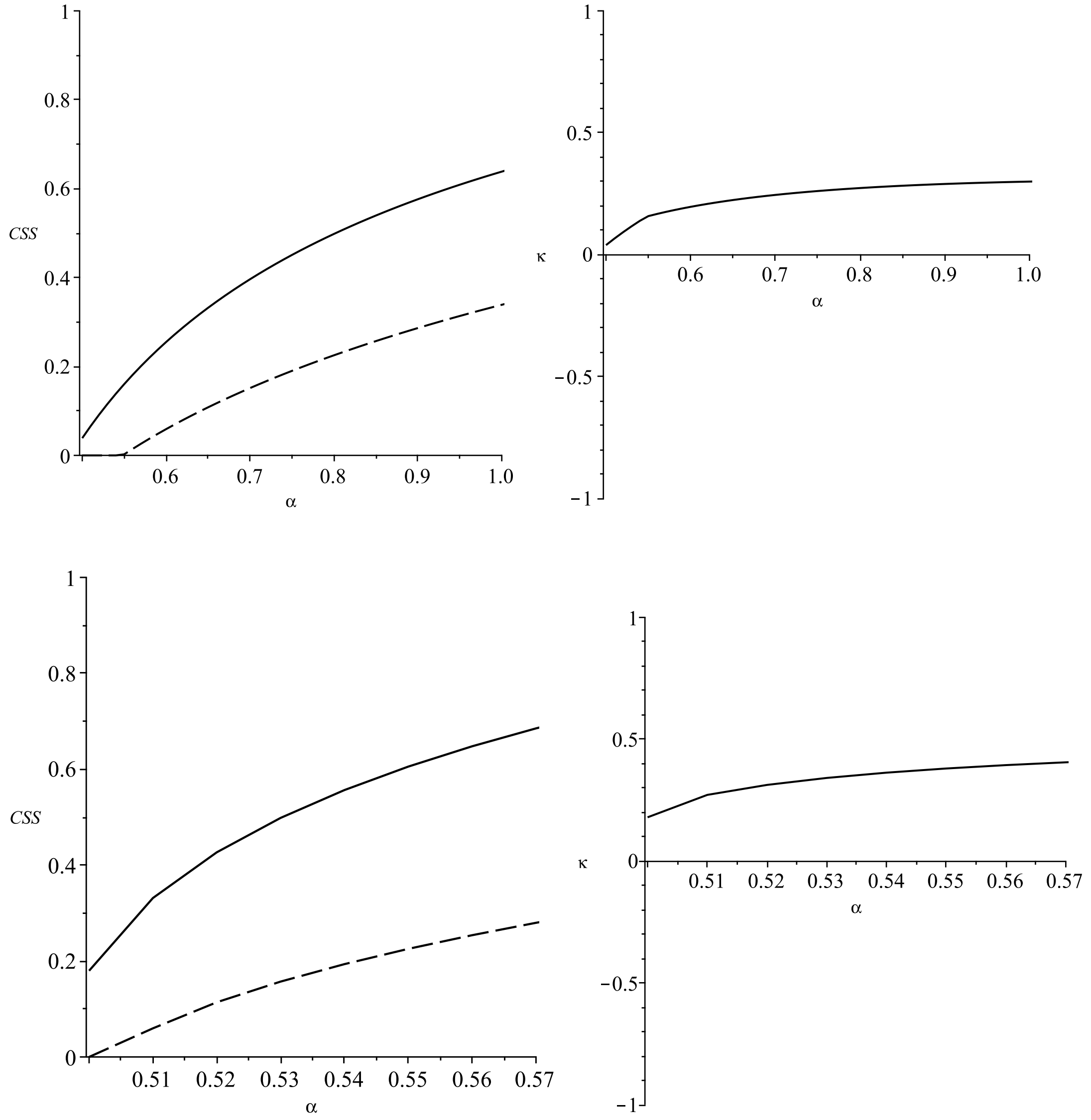

Figure S6

Plots showing investment as virulence, α, is varied for different trade-off functions to the main text, without castration (c.f. figure 1a). Top-row $k_c = k_\gamma = 0.8$ (higher curvature), bottom-row $k_c = k_\gamma = 0.6$, with parameters otherwise the same as figure 1. These plots demonstrate that a higher (lower) trade-off curvature again shifts the investment down (up) but the broad pattern remains the same. We note that taking a lower curvature in this case significantly reduces the region for which the singular point is a co-CSS.
